# Development of a novel method for the purification of C-phycocyanin pigment from a local cyanobacterial strain *Limnothrix* sp. NS01 and evaluation of its anticancer properties

**DOI:** 10.1038/s41598-019-45905-6

**Published:** 2019-07-01

**Authors:** Mahdieh Safaei, Hadi Maleki, Hamidreza Soleimanpour, Amir Norouzy, Hossein Shahbani Zahiri, Hojatollah Vali, Kambiz Akbari Noghabi

**Affiliations:** 1 Department of Energy & Environmental Biotechnology, National Institute of Genetic Engineering and Biotechnology (NIGEB), P.O. Box 14155-6343, Tehran, Iran; 2 Department of Microbiology and Microbial Biotechnology, Faculty of Life Sciences & Biotechnology, Shahid Beheshti University, Tehran, Iran; 3 Facility for Electron Microscopy Research, McGill University, 3640 Street, Montreal, Quebec, H3A 0C7 Canada

**Keywords:** Medicinal chemistry, Applied microbiology

## Abstract

C-phycocyanin (C-PC) pigment, as a natural blue dye, has particular applications in various fields. It is a water-soluble protein which has anticancer, antioxidant and anti-inflammatory properties. Here, we introduce an efficient procedure for the purification of C-PC pigment, followed by conducting a comprehensive investigation of its cytotoxic effects on human breast cancer (MCF-7) cells and the underlying mechanisms. A novel four-step purification procedure including the adsorption of impurities with chitosan, activated charcoal, ammonium sulfate precipitation, and ion exchange chromatography was employed, achieving a high purity form of C-PC with purity index (PI) of 5.26. SDS-PAGE analysis showed the purified C-PC with two discrete bands, subunit α (17 kD) and β (20 kD), as confirmed its identity by Native-PAGE. A highly purified C-PC was employed to evaluate its anticancer activity and underlying molecular mechanisms of action. The inhibitory effects of highly purified C-PC on the proliferation of human breast cancer cells (MCF-7) have detected by MTT assay. The IC50 values for 24, 48, and 72 hours of exposure to C-PC were determined to be 5.92, 5.66, and 4.52 μg/μl, respectively. Flow cytometric analysis of cells treated with C-PC, by Annexin V/PI double staining, demonstrated to induce MCF-7 cells apoptosis. Also, the results obtained from propidium iodide (PI) staining showed that MCF-7 cells treated with 5.92 μg/μl C-PC for 24 h would arrest at the G2 phase and 5.66 and 4.52 μg/μl C-PC for 48 and 72 h could induce cell cycle arrest at both G2 and S phases. The oxidative damage and mitochondrial dysfunction were evaluated to determine the possible pathways involved in C-PC-induced apoptosis in MCF-7 cells. Our findings clearly indicated that the treatment of MCF-7 cells with C-PC (IC50 for 24 h) increased the production of reactive oxygen species (ROS). Consequently, an increase in the lipid peroxidation (LPO) level and a reduction in the ATP level, mitochondrial membrane potential (MMP), glutathione (GSH) and its oxidized form (GSSG), occurred over time. The reduced expression levels of anti-apoptotic proteins, *Bcl2* and *Stat3*, plus cell cycle regulator protein, *Cyclin D1*, using Real-Time PCR confirm that the C-PC-induced death of MCF-7 human breast cancer cells occurred through the mitochondrial pathway of apoptosis. Collectively, the analyses presented here suggest that C-PC has the potential so that to develop it as a chemotherapeutic anticancer drug.

## Introduction

Cyanobacteria formerly known as blue-green algae constitute a phylum of the bacterial domain which has the ability to acquire their energy by oxygenic photosynthesis. They use special pigments to capture the light energy which differs from those of the higher plants and are called phycobiliproteins^1,2^. These unique proteinaceous compounds construct phycobilisome, a protein complex of several subunits which are embedded in thylakoid membranes. Phycobilisome is the main light-collecting complex of photosystem-II, which transmits light energy to the reaction center of photosynthesis in cyanobacteria^1–4^.

The main components of phycobilisome are bilin-containing proteins which include phycoerythrin (PE), with the maximum absorption wavelength in the range of 565 to 575 nm, phycocyanin (PC) and allophycocyanin (AC), with a maximum absorption wavelength of 620 and 650 nm, respectively. C-phycocyanin (C-PC) is the main pigment of phycobilisome complex and exists in all cyanobacterial species^1,3,5^.

C-PC is a water-soluble, non-toxic and blue colored photosynthetic pigment with an isoelectric point of about 4.5 to 5. This pigment is an oligomeric protein constructed from equal numbers of α and β subunits with the molecular weight of about 18 and 21 kDa, respectively^6^. The αβ pairs are generally arranged as a trimer (αβ)_3_ or hexamer (αβ)_6_ to build the C-phycocyanin pigment. Both α and β subunits have bilin chromophore. Bilin contains linear tetrapyrrole rings that are attached to the cysteine amino acid of the apoprotein by thioether linkages^7,8^. A different combination of various techniques, such as fractional precipitation and chromatography methods, are usually employed for purification of C-PC from a variety of cyanobacteria. Some attempts have also been made to encourage further use of chitosan and charcoal in C-PC purification process^9,10^. In general, employing the proper methods in both extraction and purification processes play a key role in the achievement of exceptionally high purity C-PC. The purity index of C-PC is determined by dividing the phycocyanin maximum absorbance wavelength to a specific absorbance wavelength of total protein; (OD_620_/OD_280_). C-PC with the purity index of above 4 supposed to have the analytical grade of purification^11,12^.

Particular properties of C- PC, as a natural blue dye, grant a series of varied functionalities to the protein which is applicable in various fields, including food and cosmetic industries, as a fluorescence probe for immunodiagnostic, and prospective therapeutic agent in oxidative stress-induced diseases^7,13–18^. Medicinal applications of C-PC arise from its anti-inflammatory, antiviral, anti-cancer, immunostimulatory and antioxidant properties^19–21^. Recent studies on the anticancer properties of C-PC exhibited a significant inhibitory effect on the growth of cancer cells in a time- and dose-dependent manner through multiple mechanisms including the induction of apoptosis, cell cycle arrest, inhibition of DNA replication and generation of reactive oxygen species (ROS)^22,23^. Prominent induction of apoptosis in cancerous cells and significantly lower toxicity on healthy cells makes it an appropriate candidate for chemotherapeutic applications (Catassi *et al*. 2006). However, the molecular mechanism involved in anticancer properties of C-PC has not been identified clearly^24^. This study was aimed to introduce and establish, for the first time, an efficient procedure for the purification of high purity C-PC pigment from cyanobacterial strains, followed by conducting more detailed investigations on its cytotoxic effects on human breast cancer (MCF-7) cells and the underlying mechanisms.

## Materials and Methods

### Isolation and identification of cyanobacterial isolate

To isolate cyanobacterial strain, sampling was performed from the swamp of National Botanical Garden of Iran and cultivated in BG11 broth at 28 °C under continuous aeration with filter sterilized air and 70 μEm^−2^s^−1^ constant illuminations through cool white fluorescent lamps. Subsequent streak plating on BG-11 agar was performed under the same light and temperature condition to attain an axenic culture of the isolate. Molecular identification of the purified *cyanobacterium* isolate was carried out by amplification and sequencing of the 16S rRNA gene with CYA106F (59-CGGACGGGTGAGTAACGCGTGA-39) and CYAN1281R (59-GCAATTACTAGCGATTCCTCC-39) as forward and reverse primers^25^. Sequence homology search in NCBI database was performed using BLAST 2.2.12. Multiple sequence alignment was carried out using ClustalW. Finally, the phylogenetic tree was constructed by the neighbor-joining method with MEGA 6.0 software.

### Purity and quantity assessment of C-phycocyanin

The purity and concentration of C-PC through the extraction and purification steps were determined, respectively, by dividing the OD_620_ to OD_280_ and by using the Equation 1, based on the equation introduced by Bennet in 1973^26^:

1 $$Concentration\,of\,C-PC=\frac{A(620)-0.474\,A(650)}{5.34}$$

C-phycocyanin content is calculated by multiplying the volume and concentration of the solution, together.

### Extraction of C-phycocyanin

#### Cyanobacterial growth and biomass harvesting

The purified cyanobacterial isolate was subcultured in BG11 broth medium under the same light, aeration and temperature conditions as mentioned in the isolation step. The growth of the isolate in a batch culture system was assessed to identify the growth phase with the highest yield of C-PC. Optical density was measured in two days interval at 750 nm and was used to plot the *cyanobacterium* growth curve. The dry weight of the total biomass in different growth phases was measured from parallel cultures of the isolate. Twenty mg dried biomass from each the lag, log, stationary and decline phases were dissolved in 1.5 ml of distilled water and subjected to multiple freeze-thaw cycles. UV-visible spectra of cell extracts were determined by centrifugation at 8000 rpm for 15 min at 4 °C and used to calculate the purity and concentration of C-PC. C-PC yield was calculated in different phases of growth, by multiplying the biomass quantity of each phase through phycocyanin concentration related to the same phase.

#### Freeze-thaw cycles

Several time-temperature combinations were used to set up a special protocol for consecutive freeze-thaw cycles, as indicated in Table 1. Efficacy of the utilized methods for C-PC extraction was compared in terms of both the C-PC purity and concentration. Various freeze-thaw protocols composed of different time-temperature combinations were carried out, and the efficiency compared by determining the purity and concentration of the C-PC in the resultant cell extract. Three consecutive cycles applied for all experiments.

**Table 1:** Comparison of different freeze-thaw methods for extracting of C-PC.

Protocol	Primary freezing	Intermediary freezing	Extended freezing	Thawing
1	—	−5 °C, 90 min	−20 °C, 3 h	+35 °C, 1 h
2	−20 °C, 10 min	−5 °C, 1 h	−20 °C, 3 h	+35 °C, 1 h
3	−20 °C, 2 min	−5 °C, 1 h	−20 °C, 3 h	+35 °C, 1 h
4	Liquid nitrogen, 2 min	−5 °C, 1 h	−20 °C, 3 h	+35 °C, 1 h
5	—	—	−70 °C, 4 h	+4 °C, 4 h
6	—	—	−20 °C, 4 h	+4 °C, 4 h

### Purification and size determination of C-PC

#### Chitosan

A stock solution of chitosan (2%) prepared by dissolving 2 g chitosan (Sigma-Aldrich, USA) in 1% acetic acid. 500 microliters of the chitosan stock solution were added to a 10 ml aliquot of cell extract, providing 0.1% chitosan/cell extract ratio with pH being adjusted to 6.5, 6.8, 6.9, 7 and 7.5. The resulted suspensions were stirred at 4 °C for 10 min and subsequently centrifuged at 8000 rpm and 4 °C for 15 min.

In another experiment, 0.25, 0.5, 1, 1.5, 2 and 2.5 ml of the chitosan stock solution were added to 10 ml aliquot of cell extract to provide the chitosan/cell extract ratios of 0.05, 0.1, 0.2, 0.3, 0.4 and 0.5%, respectively. PH was adjusted to 6.9, and stirring plus centrifugation performed in the same condition.

#### Activated charcoal

Activated carbon was added at the final concentration of 8, 10, 12, 14 and 16% to the extract and stirred at 4 °C for 10 min followed by 15 min centrifugation at 8000 rpm (at 4 °C).

#### Ammonium sulfate precipitation

Salting out with ammonium sulfate (AS) was used to achieve further purification of phycocyanin. To find out at what AS saturation the C-PC protein gets precipitated, increasing concentrations of ammonium sulfate were added to equal amounts of the cell extract and stirred gently at 4 °C for 5 hours, followed by centrifugation (15 min, 4 °C). Then, the supernatant was discarded and the precipitate was dissolved in sodium phosphate buffer (10 mM, pH 7.5) and the absorption spectrum of both the supernatant and dissolved precipitate was measured in the wavelength range 200–700 nm. The absorbance spectra were compared to find out the most profitable concentration and effective ammonium sulfate fractionation procedure for the purification of C-PC.

#### Anion-exchange chromatography

The dissolved precipitate (from the previous step) was dialyzed against phosphate buffer (10 mM, pH 7.5) overnight at 4 °C and loaded onto a 1.2 × 10 cm column which was packed with Q-Sepharose XL (GE Healthcare) anion exchange resin and equilibrated with the same buffer of dialysis. An elution flow rate of 1 ml/min was performed with a multiple-slope gradient of 0 to 0.5 M NaCl solution in phosphate buffer (10 mM, pH 7.5) according to the program stated in Table 2. Fractions of 1 ml were collected and their absorption spectra were acquired over a wavelength range of 200–700 nm. The purity and concentration of phycocyanin (in the eluted fractions) were calculated using the obtained spectra. Fractions with the purity index above 4 were freeze-dried (after pooling and dialyzing against distilled water). The pooled fractions was lyophilized and used for subsequent bioassays and analytical tests.

**Table 2:** Multi-gradient elution program used for purification of C-PC by ion exchange chromatography method.

Time(min)	Buffer A (%)	Buffer B (%)
0	95	5
30	95	5
130	85	15
270	83	17
335	70	30
400	0	100

#### Phycocyanin size determination

The molecular weight of the purified C-PC was determined using sodium dodecyl sulfate polyacrylamide gel electrophoresis (SDS-PAGE). Native PAGE protein was prepared in a non-reducing and non-denaturing sample buffer as well. Coomassie brilliant blue and silver nitrate were used for gel staining.

### Evaluation of anticancer specificity of C-PC

#### Cell culture and MTT assay

DMEM (Dulbecco’s Modified Eagle Medium) medium containing 10% fetal bovine serum (FBS) was used for cultivation of human breast cancer cell-line (MCF-7) and evaluation of anti-proliferation properties of C-PC. The cells were seeded at the cell density of 2 × 10^3^ cells/well on to 96-well microplate. C-PC was dissolved in DMEM to make a stock solution of 0.1%. Various concentrations of C-PC (0, 0.4, 2, 4, 5, 6, 7, 8, 9, 10 and 12 μg/μl) were established within the consecutive wells (in a total volume of 100 μl culture medium). The effect of various C-PC concentrations was assessed in 24, 48 and 72 hour time periods separately. Cultivation of MCF-7 cells was performed in an atmosphere of 5% CO_2_ and 95% humidity at 37 °C.

Upon ending the particular time of the treatment, 10 μl of 0.5% MTT (Sigma-Aldrich, USA) solution in PBS was added to each well and incubated for an additional 4 h at 37 °C. The resulted purple-blue formazan precipitate was dissolved in 100 μl of a 10% SDS (in 0.01 M HCl solution) and viability of the treated cells were assessed indirectly by measuring the absorbance at 580 nm wavelength, considering the absorbance at the reference wavelength of 692 nm using a microplate reader (Titertek multiskan microplate reader, LabSystems Multiskan, Roden, Netherlands). The half maximal inhibitory concentration (IC_50_) was determined for each exposure time, separately.

In the next step, MCF-7 and human primary fibroblast cell lines were treated similarly with the IC_50_ concentration of C-PC for 24, 48 and 72 h to compare the C-PC effect on healthy and cancerous cells. The experiments were repeated three times.

#### Cell membrane integrity and viability assay using FDA/PI staining method

The effect of C-PC on the membrane integrity of the cancerous cells was evaluated by fluorescent microscopy analysis of fluorescein diacetate (FDA) and propidium iodine (PI)-stained cells. The cells at the same density, as in the previous experiments, were cultivated for 24 h in the presence of the related IC_50_ concentration of C-PC. At the end of incubation period, 100 μl of FDA solution was added into the wells and the plate was then kept in the dark at room temperature for 10 min before fluorescence microscope imaging (excitation wavelength 493 nm; emission wavelength 510 nm) of live cells. After that, 30 μl of PI solution was added into the wells and the images was taken while holding the plate in the dark at room temperature for 10 min. FDA staining solution was prepared by adding 40 μl of FDA to 10 ml of 0.5% acetone solution in phosphate buffered saline (PBS). PI was dissolved in PBS to provide the 0.002% stain solution.

#### Apoptosis assay

MCF-7 cells were seeded in 6-well plates with a density of 7 × 10^4^ cells/ml. The cells were treated at particular IC_50_ concentrations of C-PC in corresponding exposure time of 24, 48 and 72 h. Flow cytometry was performed to detect the resulting apoptotic MCF-7 cells. At the end of incubation period, the culture medium was removed and cells were washed according to the instructions provided by Annexin V/PI kit. Cell dissociation was carried out through trypsinization. Then, 200 μl of 2× Annexin V binding buffer and 1 μl Annexin were added to the detached cells in each well and then placed in the dark at room temperature for 15 min. Flow-cytometry was performed after addition of 0.3 μl of PI (1 mg/ml) per sample (FACSCalibur; BD 6Bioscience, USA), and the results were analyzed by Flow Jo software version 10.1.

#### Analysis of cells cycle arrest

MCF-7 Cells were cultured and treated under the same conditions as in the previous experiment. The medium was removed upon termination of the exposure time and cells were detached by trypsinization. Then, the cells were fixed with 70% cold ethanol while standing overnight at 4 °C. The cells were washed with PBS and resuspended in 500 μl staining buffer composed of 10 μg/ml propidium iodide (PI), 100 μg/ml RNase A and 0.1% (v/v) Triton X-100. Cell cycle analysis was performed by flow cytometry and the results were analyzed by Flow Jo software version 10.1.

#### Determination of GSH and GSSG content

The different levels of reduced glutathione (GSH) and its oxidized form (GSSG) was determined in MCF-7 cells to evaluate the oxidative state of the cells after 24, 48 and 72 hours exposure to IC_50_ concentrations of C-PC. GSH and GSSG contents were measured by spectrofluorimetry using O-phthalaldehyde (OPA) and N-ethylmaleimide (NEM) probes based on the method developed by Hissin and Hilf (1976)^27^.

#### ROS and MMP determination

MCF-7 cells (7 × 10^4^ cells/well) were treated with C-PC concentration of 5.92 μg/μl (IC50/24 h) for 24 h. Dichloro-dihydro-fluorescein diacetate **(**DCFH-DA) was used to evaluate the effect of C-PC on intracellular reactive oxygen species (ROS) induction. For this purpose, the MCF-7 cell pellet was exposed to 10 mM DCFH-DA, incubated at 37 °C for 30 min and dissolved in phosphate buffered saline (PBS). The fluorescent cationic dye, rhodamine 123 (10 mM), was used to determine the mitochondrial membrane permeability (MMP). Finally, the stained cells were analyzed by flow cytometer using flowing software (Ver 2.5-1), equipped with a 488 nm argon ion laser. The fluorescence signals (for at least 10000 counts per sample) were obtained using a 530 nm band pass filter (FL-1 channel). To detect of ROS and MMP, the cells were read in a flow cytometer (FACS Calibur, BD Bioscience, USA).

#### Determination of lipid peroxidation (Malonyl dialdehyde)

The level of lipid peroxidation (malonyl dialdehyde, MDA) was examined as described by Zhang *et al*.^28^ by the treatment of MCF-7 cells (7 × 10^4^ cells/well) with the IC50 concentration of C-PC for 24, 48 and 72 h. The amount of 0.25 ml of 0.05 M sulfuric acid was added to 0.2 ml of cell fractions. After addition of 0.3 ml of a solution containing 0.2% Thiobarbituric acid (TBA), all the samples are placed in a boiling water bath for 30 minutes. Subsequently, the samples are transferred to an ice bath and n-butanol (0.4 ml) is added to each sample. The samples were centrifuged at 3500 × g for 10 min and the absorbance was read at 532 nm with an ELISA reader (Tecan, Rainbow Thermo, Austria).

#### Determination of ATP level

MCF-7 cells (7 × 10^4^ cells/well) were treated by the IC50 concentration of PC for 24, 48 and 72 h, and the ATP levels (in mitochondria) measured by bioluminescent somatic cell assay kit (sigma Aldrich.MO 63103, USA).

#### Real-Time PCR analysis

MCF-7 cells (7 × 10^4^ cells/well in 6-well culture plates) were exposed to 5.92 μg/μl (IC50/24 h) C-PC for 24 h. Total RNA was extracted using RNAx plus (Sinaclon, Iran) solution according to the company protocol. The quality and quantity of extracted RNA were evaluated by electrophoresis and Nano-Drop. The synthesis of cDNA was performed using the RevertAid First Strand cDNA Synthesis Kit (Thermo Scientific, USA) in accordance with the factory instrumentation protocol. The primers for the *Cyclin D1* (cell cycle regulator), *Bcl2* (apoptosis regulator) and *Stat3* (the transcription factor, signal transducer and activator of transcription-3) genes were designed by using the NCBI genomic sequences and the Oligo 6 software. Glyceraldehyde-3-phosphate dehydrogenase (*GAPDH*) was used as a housekeeping gene (Table 3). The PCR was performed using SYBER Green Real-Time PCR master mix (Ampliqon, UK) with the Riley Timing Machine (Corbett, Germany). The PCR was performed to make a final volume of 10 μl containing 5 μl of fast start master solution, 0.3 μl of each primer and 1 μl of cDNA. Amplification was performed as follows: initial denaturation at 95 °C for 15 min; 40 cycles of denaturation at 95 °C for 30 s, annealing at 60 °C for 30 s and extension at 72 °C for 20 s; and a final extension at 72 °C for 10 min.

**Table 3:** Primer sequences used for RT-PCR analysis.

Gene	Primer	Sequence (5′− > 3′)	Product size
*GAPDH*	Forward primer	CTCA TGACCACAGTCCATGC	155bp
*GAPDH*	Reverse primer	TTCAGCTCTGGGATGACCTT GGAGAAGGACATCAGCGGTAAGAC	
*STAT3*	Forward primer	AGAGATAGACCAGTGGAGACACCAG ATCAAGTGTGACCCGGACTGCCT	149bp
*STAT3*	Reverse primer	ACGTCGGTGGGTGTGCAAGC	
*Cyclin D1*	Forward primer	CTGCACCTGACGCCCTTCACC	161bp
*Cyclin D1*	Reverse primer	CACATGACCCCACCGAACTCAAAGA	
*BCL2*	Forward primer	CTGCACCTGACGCCCTTCACC	119bp
*BCL2*	Reverse primer	CACATGACCCCACCGAACTCAAAGA	

### Statistical analysis

All experiments were repeated at least three times, and the results were reported as mean ± S.D. To statistical analysis, the results were subjected to one-way ANOVA using Sigma Plot Version 12.0. A value of P < 0.05 was considered as Significant. In the RT-PCR, each reaction of cDNA synthesis was evaluated using LinReg software and real-time PCR data analysis was done by Rest 2009 software.

## Results

### Phylogenetic analysis and features of the isolated strains

**Figure 1:**
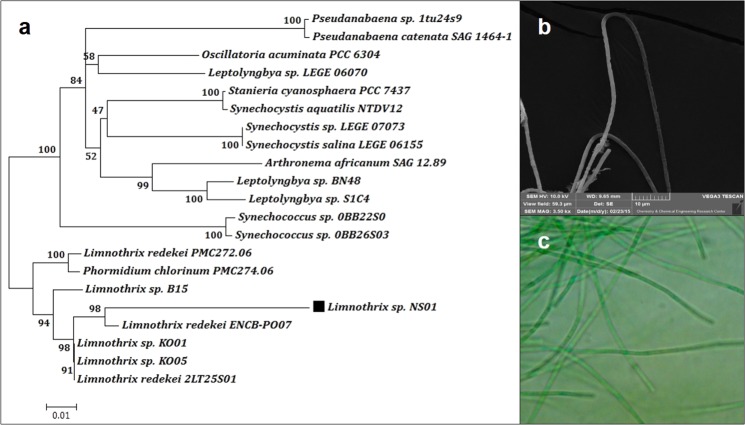
Phylogeny and morphology of *Limnothrix* sp. NS01. (**a**) Phylogenetic tree based on 16S rRNA gene sequencing, (**b**) Light microscopy image, (**c**) SEM image.

Initial morphological analysis of cyanobacterial strain using light microscopy and scanning electron microscopy (SEM) revealed the presence of filamentous structures. Homology searching and phylogenetic analysis of the 16S rRNA gene sequence revealed that the cyanobacterial strain had 99% identity with the reference sequences of the members of the genus *Limnothrix*. The strain NS01 recognized as belonging to genus *Limnothrix*. The amplification of 16S rRNA gene segment (1260 bp) from cyanobacterial strain was achieved and phylogenetic analysis was done based on the BLAST search and multiple sequence alignment using CLUSTAL-W and MEGA-6 software. The phylogenetic tree was constructed by subjecting the aligned sequence to neighbor-joining with 1000 times bootstrap replication and a substitution model p-distance (Fig. 1). The 16S rRNA gene sequence of the isolate was submitted to NCBI and registered with the name of *Limnothrix* sp. NS01, through accession numbers of KX656895.

### Determination of optimal time for phycocyanin extraction

**Figure 2:**
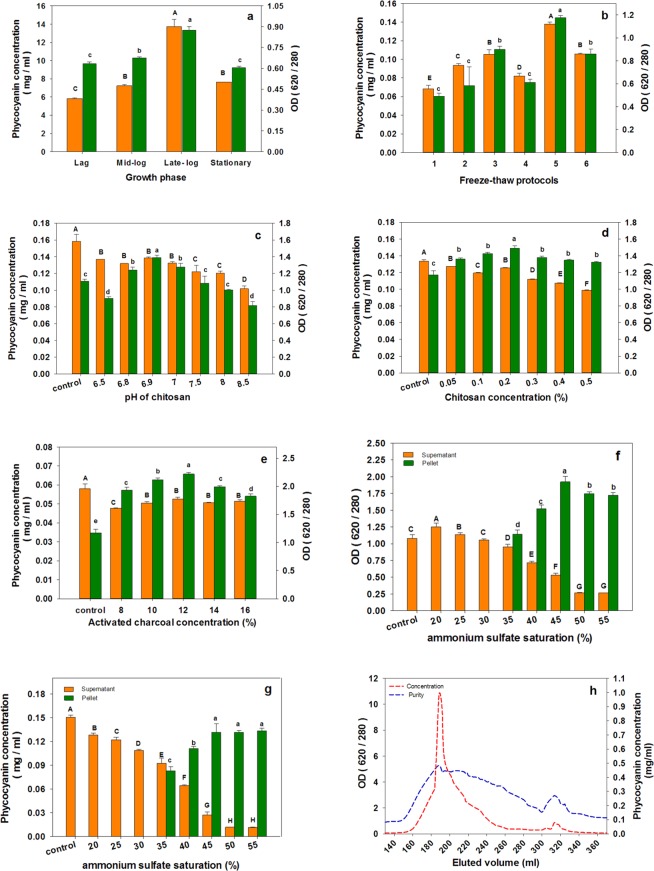
Purity and concentration of C-PC in various extraction and purification steps and experiments. (**a**) Different growth phases of the organism, (**b**) Different freeze-thaw protocols for C-PC extraction, (**c**) The effect of pH on chitosan affinity precipitation of impurities (chitosan concentration; 0.2% w/w), (**d**) The effect of chitosan concentration on purification of C-PC (pH 6.9), (**e**) The effect of activated charcoal concentration, (**f**) The effect of ammonium sulfate saturation extent on purity of the phycocyanin in the precipitate and supernatant, (**g**) The effect of ammonium sulfate saturation extent on concentration of the phycocyanin in the precipitate and supernatant, (**h**) Purity and concentration of C-PC in the eluted fractions through ion exchange chromatography; Values represented as mean ± SD. n = 3; Different small letters and different capital letters above the bars represent statically significance difference in purity and concentration of C-PC respectively.

Analysis of the biomass samples taken every 2 days interval exhibited a concomitant increase in both the purity and concentration of C-PC by increasing the incubation time, reaching to its maximum at the late-logarithmic phase. Further incubation could neither increase purity nor concentration of the C-PC (Fig. 2a). The highest C-PC content and purity were achieved by three cycles of four hours freezing at minus 70 °C after thawing in the refrigerator (for three hours) (Fig. 2b).

### Purification of C-PC from *Limnothrix* sp. NS01

#### Chitosan and activated charcoal

The cell extract was adjusted for pH within a range of 6.5 to 8.5, where the chitosan is partially soluble and has a positive net charge. As shown in Fig. (2c), the pH 6.9 was the most efficient pH in efficient depletion and clearance of impurities from the cell extract by chitosan. In searching for the most efficient concentration of the chitosan in depletion of impurities, the chitosan/cell extract ratio of 0.2% was found to be the most appropriate quantity of chitosan (Fig. 2d). The most appropriate concentration of activated charcoal, considering both the yield and purity of C-PC was 12%. The higher amounts of charcoal resulted in both lower purity and concentration of the PC (Fig. 2e).

#### Ammonium sulfate precipitation

Based on the results of different percentages of ammonium sulfate (AS), the purity index of non-precipitated C-PC in the solution was gradually decreased by increasing the AS concentrations over the 20% while the purity index of the precipitated C-PC showed an increase up to the 45% AS concentration followed by a steady reduction in purity with the higher AS concentrations. As shown in (Fig. 2f,g), ammonium sulfate precipitation at 45% w/v saturation was found to be the most appropriate concentration, achieving the highest purity and a notable yield of the C-PC.

#### Purification by ion-exchange chromatography

As a final purification step, Q-Sepharose ion exchange chromatography was used to obtain a high purity C-PC pigment. Regarding the elution program indicated in Table 2 and based on the chromatography results, C-PC left the column when NaCl concentration in the eluent reaches to 80 mM (in 10 mM sodium phosphate buffer pH 7.5). The amount of the purified C-PC with the purity index of above 4 was 85.31% of the total eluted C-PC. It was calculated by subtracting the area under the plot of the highly pure fractions from the entire area under the C-PC related peak (Fig. 2h). All fractions containing C-PC were pooled, resulting in a purity index of ~4.7 and the most purified C-PC, in this step, had a purity index of about 5.26. The second peak which was eluted after the C-PC peak was related to allophycocyanin and left the column with NaCl concentration of the eluent reached to about 125 mM. Allophycocyanin with specific absorbance at 650 nm gave rise to 620/280 absorbance ratio upon leaving the column.

#### Determination of molecular weight

**Figure 3:**
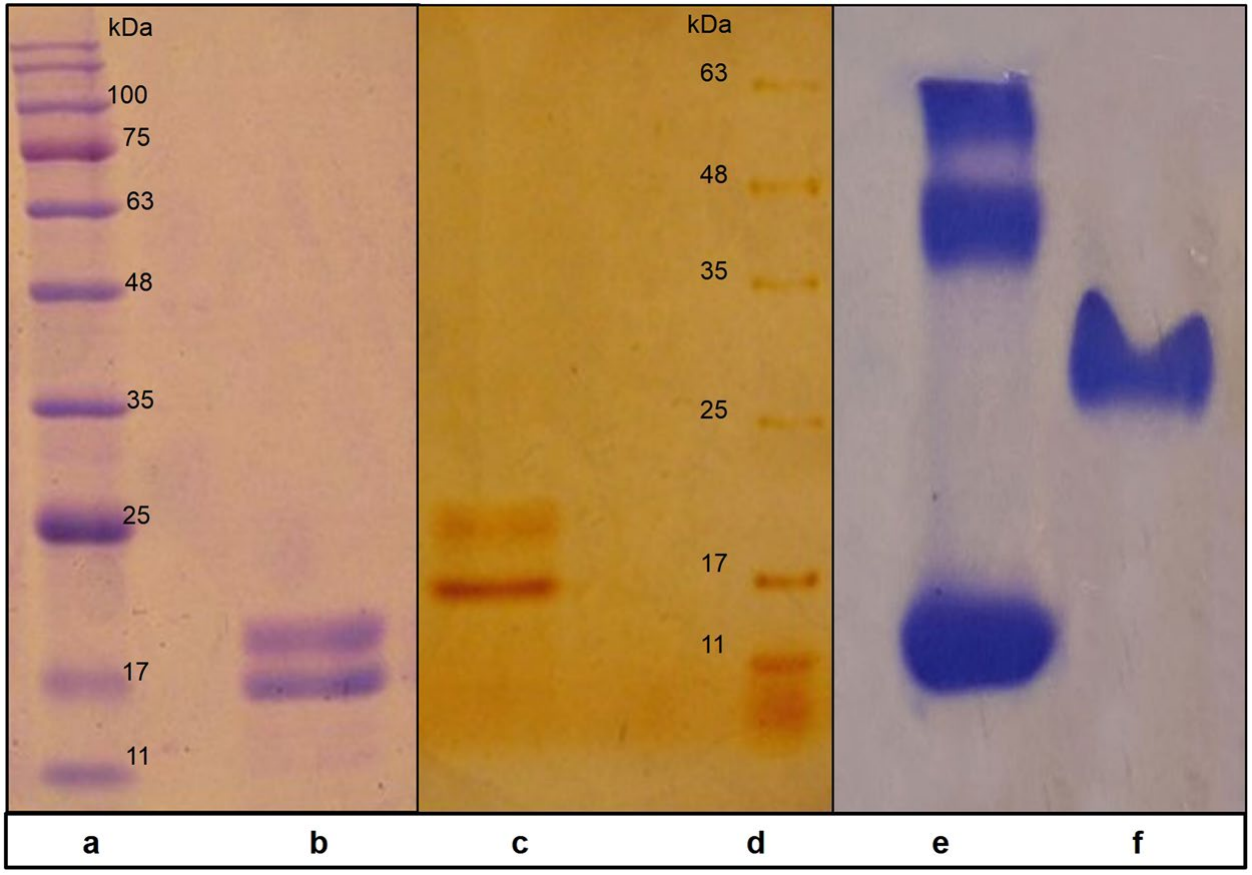
Native and SDS-PAGE profiles of the purified C-PC from *Limnothrix* sp. NS01. (**a**) Protein marker, (**b**) SDS-PAGE bond of C-phycocyanin stained with Coomassie brilliant blue, having a molecular weight of about 17 and 20 kD, related to α and β subunits, respectively, (**c**) SDS-PAGE bond of C-phycocyanin stained with silver nitrate, (**d**) Protein marker, (**e**) Blue-native PAGE of bovine serum albumin (BSA) as a protein marker, (**f**) Single bond of the purified C-PC by Blue-native PAGE.

The results obtained from SDS-PAGE with either Coomassie brilliant blue or silver nitrate staining showed two bonds corresponding to α and β subunits of *Limnothrix* sp. NS01 phycocyanin with molecular weights of about 17 and 20 kDa, respectively. In addition, the results from native polyacrylamide gel electrophoresis (Native-PAGE) for C-PC sample demonstrated a single bond which was in agreement with spectrophotometric results, indicating the high purity of C-PC product (Fig. 3).

**Figure 4:**
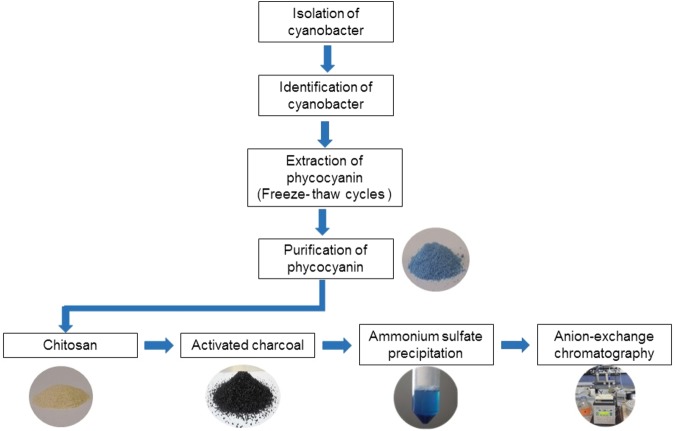
Sequential steps of C-PC purification from *Limnothrix* sp. NS01.

Sequential steps of C-PC purification from the isolated cyanobacterial strain were illustrated in Fig. (4).

### Anticancer properties of C-PC

#### Inhibitory effect of C-PC on the growth of MCF-7 cells

**Figure 5:**
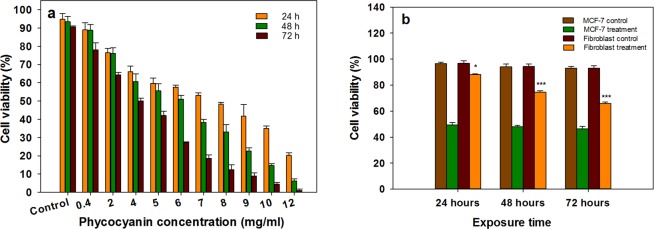
Cell proliferation measurement by MTT colorimetric assay, (**a**) Viability of MCF-7 cells exposed to various C-PC concentrations for 24, 48 and 72 h, (**b**) Viability of MCF-7 and human fibroblast cells after treatment with C-PC at the related IC50 concentrations for 24, 48 and 72 h. Values represented as mean ± SD. n = 3. *P < 0.05, ^***^P < 0.001 compared with control.

The MTT assay was performed to examine the cytotoxic effect of C-PC on MCF-7 cells and the results showed that C-PC affects the cells in a time and dose-dependent fashion. An increase in either concentration or exposure time of C-PC led to the stronger inhibition of the cells growth. As shown in Fig. 5(a), the IC_50_ values of C-PC were found to be 5.92, 5.66 and 4.52 μg/μl for 24, 48 and 72 hours exposure, respectively. C-PC had no significant toxicity on human fibroblast cells at the IC_50_ concentrations (after 24, 48 and 72 hours of exposure) (Fig. 5b).

#### Analysis of the integrity and functionality of the cell membrane by FDA and PI uptake method

**Figure 6:**
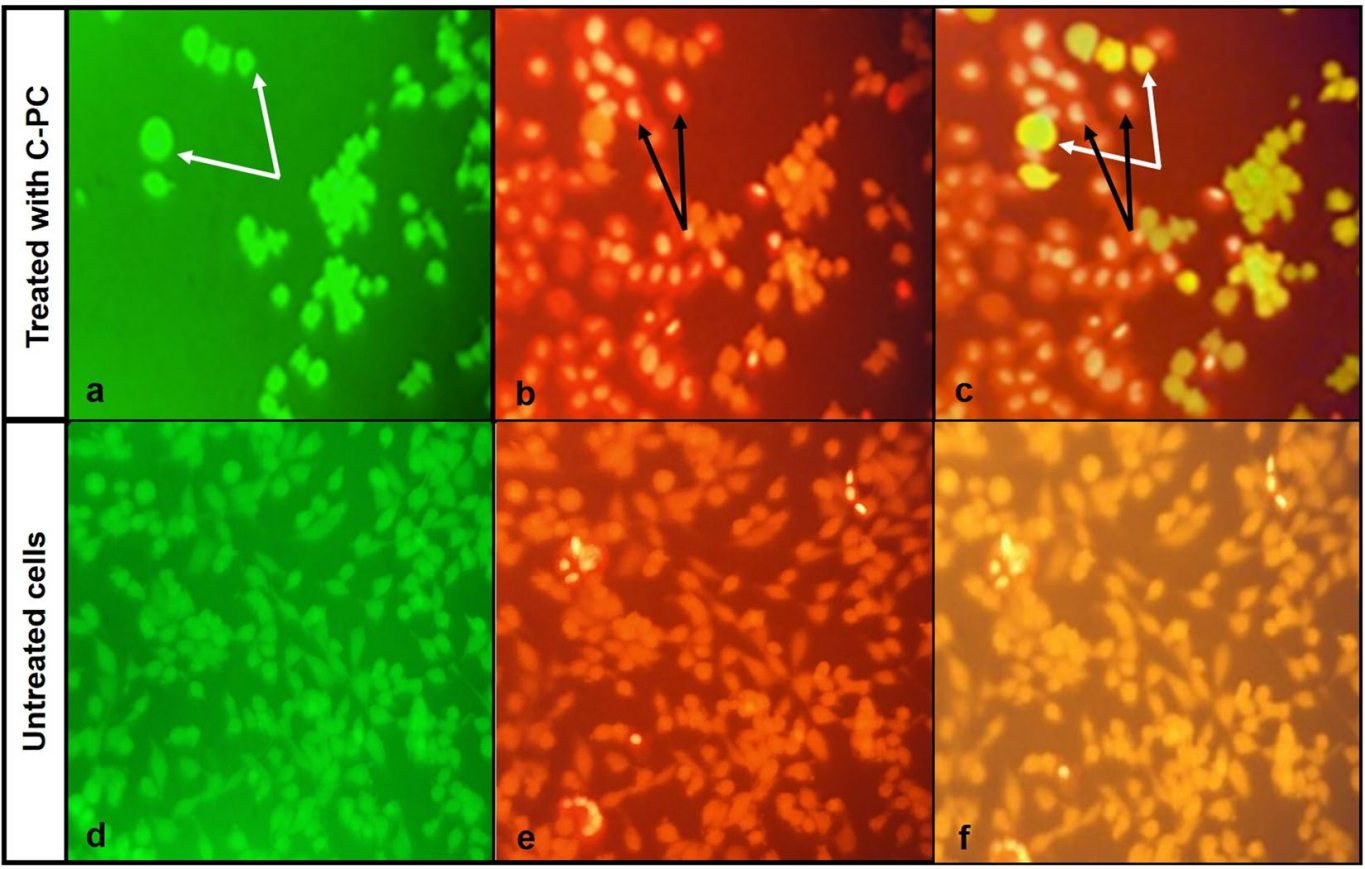
Fluorescent microscopy images of MCF-7 cells stained with FDA and PI. (**a**,**d**) Live cells which are stained by FDA; (**b**,**e**) Dead cells which are stained by PI (after staining with FDA); (**c**,**f**) Merged picture of FDA and PI staining images, white and black arrows represent live and dead cells respectively.

Fluorescein with a green fluorescence is produced from the non-fluorescent FDA by the function of the cytoplasmic esterase and acts as an indicator of cell viability. On the other hand, PI with a red fluorescence get access to cell DNA just through a nonintegrated cell membrane. Fluorescent microscopy assessment by FDA and PI is a suitable method for simultaneous staining of the live and dead cells together. FDA/PI staining showed a considerable reduction in live/dead cells ratio in MCF-7 cells treated with the IC_50_ concentration of C-PC (Fig. 6).

#### Induction of apoptosis in human breast cancer cell line (MCF-7) by C-PC

**Figure 7:**
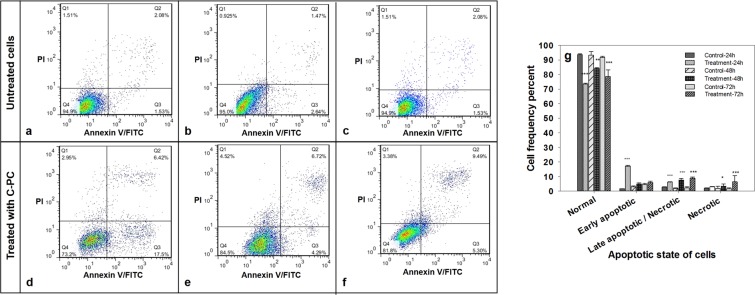
Effects of C-PC exposure (at the related IC50 concentration) on apoptosis induction in MCF-7 cells. (**a**–**c**) The cultivation of MCF-7 cells for 24, 48 and 72 hours (as controls), (**d**–**f**) MCF-7 cells treatment with C-PC for 24, 48 and 72 hours, respectively, (**g**) frequency percent of C-PC treated and untreated cells residing in different physiological states after 24, 48 and 72 hours of experiment. Values represented as mean ± SD. n = 3. *P < 0.05, ^***^P < 0.001 compared with control.

The results of Annexin V-FITC/PI staining by flow cytometric analysis showed a higher rate of early apoptotic cells treated with the related IC_50_ concentrations of C-PC, detecting 24 hours after start of exposure. The longer the exposure time, the more apoptotic cells will detect, followed by outnumbering apoptotic cell over non-apoptotic ones, as a result of encountering the late apoptotic state (Fig. 7).

#### Effect of C-PC on cell cycle arrest in human breast cancer MCF-7 cells

**Figure 8:**
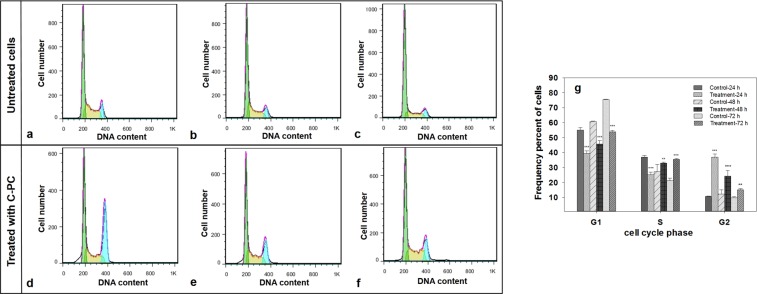
Influence of C-PC on the cell cycle progression of MCF-7 cells. (**a**–**c**) untreated MCF-7 cells after 24, 48 and 72 hours of experiment, (**d**–**f**) C-PC treated MCF-7 cells for 24, 48 and 72 hours at the related IC50 concentrations, (**g**) frequency percent of C-PC treated and untreated cells residing in the various cell cycle phases. Values represented as mean ± SD. n = 3. ^**^P < 0.01, ^***^P < 0.001 compared with control.

The MCF-7 breast cancer cells treated with C-PC over 24 h resulted in a considerable increase in the proportion of cells in G2/M phase and decreased percentage of MCF-7 cells in S and G1 phases. Longer exposure would increase the number of cells in both S and G2 phases but to a lower extent. Accordingly, the results imply the changes in the requisite checkpoint mediated cell cycle arrest in response to C-PC over time (Fig. 8).

#### Determination of glutathione (GSH) and glutathione disulfide (GSSG) contents

**Figure 9:**
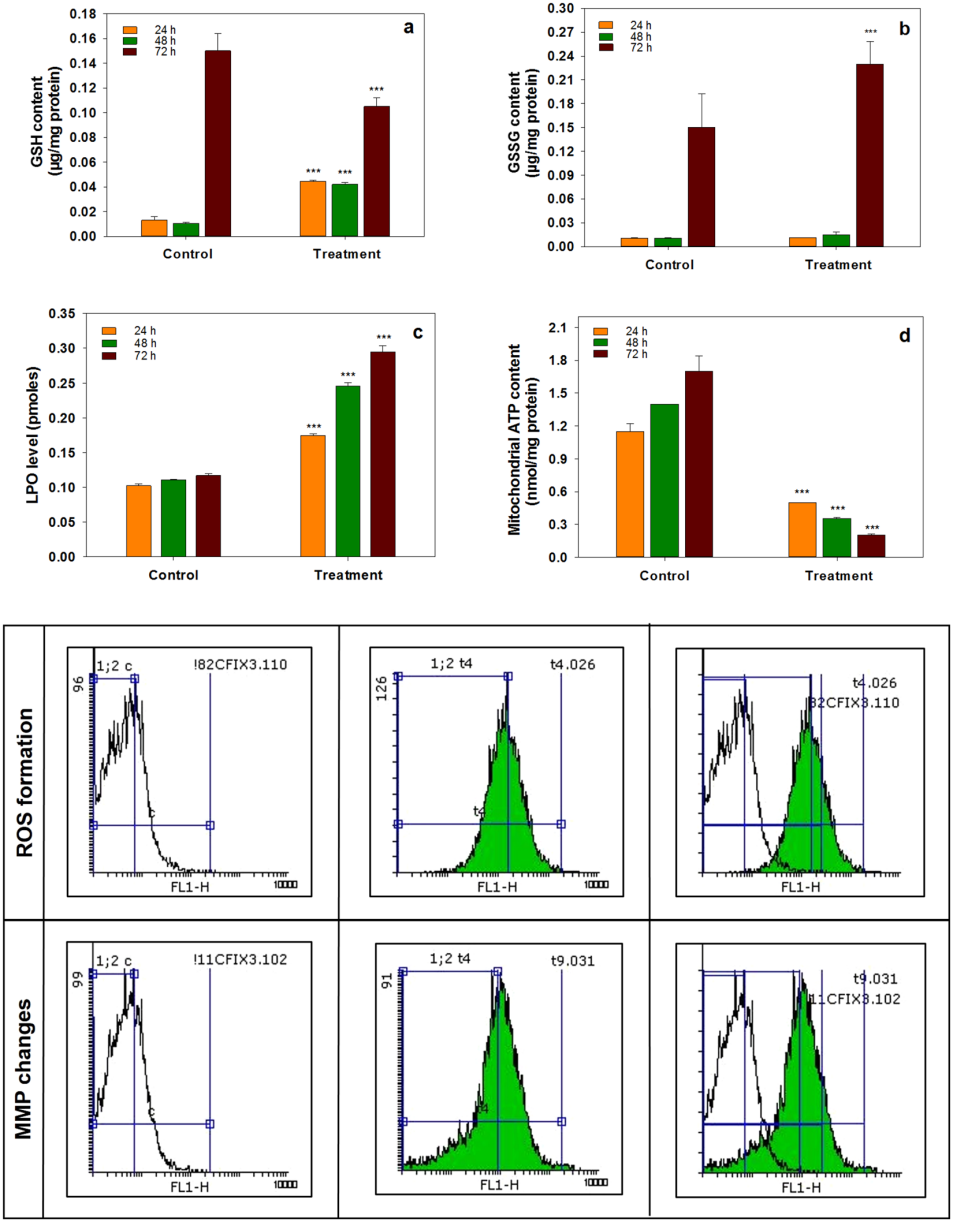
Oxidative state of MCF-7 cells. (**a**) Mitochondrial GSH content of untreated and C-PC treated MCF-7 cells for 24, 48 and 72 hours at the related IC50 concentrations, (**b**) Mitochondrial GSSG content of untreated and C-PC treated MCF-7 cells for 24, 48 and 72 hours at the related IC50 concentrations (**c**) Lipid peroxidation level in untreated and C-PC treated MCF-7 cells for 24, 48 and 72 hours at the related IC50 concentrations, (**d**) Mitochondrial ATP level in untreated and C-PC treated MCF-7 cells for 24, 48 and 72 hours at the related IC50 concentrations. Values represented as mean ± SD. n = 3. ^***^P < 0.001 compared with control.

Intracellular glutathione concentration in both reduced (GSH) and oxidized (GSSG) forms were evaluated by spectrofluorometric method after incubating the MCF-7 cells with the IC50 concentration of C-PC for 24, 48 and 72 hours. GSH is one of the most important ROS eliminating factors. The different levels of GSH and GSSG ratio were detected to evaluate the oxidative state of the cells. Results showed a progressive reduction in the GSH/GSSG ratio by increasing time since exposure to C-PC (Fig. 9a,b).

#### Effect of C-PC on lipid peroxidation (LPO) and ATP level

Since the increase in ROS production can lead to cell membrane damage through the chain reaction mechanism of oxidative degradation of lipids, we examined the level of LPO in MCF-7 cells after incubation with C-PC, at particular IC50 concentrations, in corresponding exposure time of 24, 48 and 72 h. Our results showed that C-PC induces the production of ROS in MCF-7 cells and there occur an increase of LPO levels in C-PC-induced MCF-7 cells after 72 h at a higher level than 24 and 48 hours of C-PC treatment (Fig. 9c). The increase in LPO levels can result in disruption of mitochondrial electron transport chain function and thereby mitochondrial ATP production. Consequently, ATP levels were measured and the analysis was revealed that the mitochondrial ATP levels decreased after incubation with C-PC (at IC50 concentrations) at the 24-, 48- and 72- hour test periods in a time-dependent manner (Fig. 9d). The reduction in ATP levels was significant at 72 hours compared with those treated with C-PC at 24 and 48 hours.

#### Effect of C-PC on mitochondrial reactive oxygen species and mitochondrial membrane potential *(Δψm)*

The effect of C-PC on mitochondrial production of reactive oxygen species (ROS) in the MCF-7 cells at a concentration of 5.92 μg/μl for 24 hours was assayed to further understand the possible anticancer mechanism of C-PC. According to the results obtained from flow cytometry data analysis, shifting the significant peak indicates that high levels of ROS produced by the MCF-7 cells after exposure to C-PC (Fig. 9e). The increase in the ROS production would induce ROS-associated damages to DNA, proteins lipids and cell function, ultimately leading to apoptosis signaling cascades. Since mitochondria are the main source of ROS production in the cells, there should be an association between the level of ROS and mitochondrial membrane potential (MMP). Therefore, we studied the influence of C-PC on MMP. Flow cytometry data analysis showed that the MMP was declined when MCF-7 cells was incubated with C-PC at a concentration of 5.92 μg/μl for 24 h.

#### Effect of C-PC on transcription profile of Bcl-2, Cyclin D1, Stat3 genes

**Figure 10:**
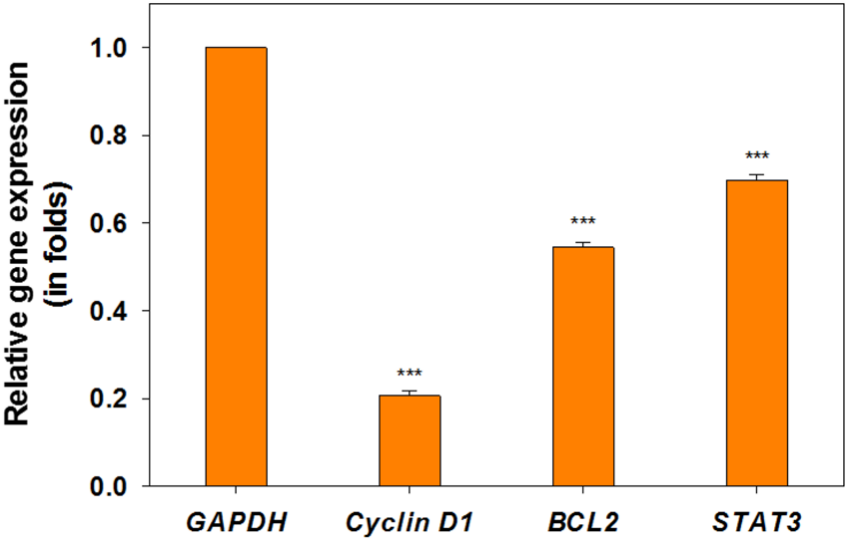
Effects of C-PC on the expression of *Bcl2*, *Stat3* and *Cyclin D1* genes in MCF-7 cells. Values represented as mean ± SD, n = 3. ^***^P < 0.001 compared with control.

Real-Time PCR was used to analyze the transcription profiles of *Bcl2*, *Stat3* and *Cyclin D1* genes in C-PC-induced MCF-7 cells after 24 hours. Glyceraldehyde-3-phosphate dehydrogenase *(GAPDH*) gene (as a common housekeeping gene used in comparisons of gene expression data) used as internal control for quantitative gene expression analysis. As shown in Fig. (10), the expression of *Bcl2*, *Stat3* and *Cyclin D1* coding mRNA in MCF-7 cells treated with C-PC is reduced compared to the control subjects. Changes in expression levels of *Bcl2*, *Stat3* and *Cyclin D1* genes in MCF-7 cells treated with C-PC indicated a decrease in expression of three genes as 1.83, 1.45 and 4.85 times, respectively, in comparison with the control normal cells.

## Discussion

It is worth mentioning that there has not yet been a well-established specification and standard method for extraction and purification of C-PC form cyanobacteria due to the remarkable diversity of methods. Therefore, the design of an effective purification strategy to obtain a high purity form of C-PC, having an adequate commercial value, is of great importance. Several methods including aqueous two-phase extraction, ammonium sulfate precipitation, charcoal and chitosan adsorption, membrane filtration, gel filtration, hydrophobic interaction chromatography, and ion exchange chromatography have frequently been used to purify c-phycocyanin^29^. Obviously, it’s inevitable to combine several methods in order to have a highly purified form of C-PC. Inefficient C-PC extraction, an improper combination of purification methods, inappropriate order of the purification steps, and unsuitable or far-optimal condition may encounter a failure in obtaining a highly purified form of C-PC with reasonable yield.

In this study, C-PC was extracted properly, and the optimal conditions were determined to remove a considerable part of the impurities by providing a relative advantage of chitosan and charcoal. Then, C-PC was further purified using a proper ammonium sulfate concentration. Ion exchange chromatography was employed as the key ultimate step in the purification of C-PC, using a multiple-slope gradient elution program, while C-PC was eluted from the column and resulted in a highly purified form with a purity index of 5.26. The extraction efficiency of the C-PC, as the first step in the harvesting of this pigment from cyanobacterial biomass, is a challenging task. A high amount of the biomass besides higher C-PC contained in the cell directed to the late logarithmic phase as the best growth phase for harvesting biomass. In this study, various combinations of different hypothermal steps were examined in consecutive freeze-thaw cycles to evaluate the impact of freezing conditions on the yield and purity of the released C-PC from the ruptured cells. Extracted C-PC was subjected to chitosan and charcoal treatments, AS precipitation and ion exchange chromatography to achieve further purified form of C-PC. Chitosan is quite soluble at pH values below 6.5^30^. Partially hydrated chitosan in the test pH range forms tiny swollen positively charged granules which separated along with the adsorbed impurities by centrifugation. As C-PC has a negative charge in the above-mentioned pH, it competes with other proteins and organic impurities for adsorption sites on chitosan granules. The amount of chitosan affects either purity or yield of C-PC in the supernatant. The pH 6.9 and chitosan concentration of 0.2% found to be the most appropriate condition for the removal of protein impurities. The higher amount of chitosan resulted in low yield and also the low purity of C-PC. Highly porous structure of activated charcoal provides a better specific surface area and rich miscellaneous aromatic groups for hydrophobic binding of substances with low effective charge^30^. Charcoal, exhibited the fine pore structure, make a size preference to the adsorbing substances with lower molecular weight because of higher accessible binding sites. The employment of charcoal at a pH value away from the isoelectric point (pI) of C-PC led to an enhancement of the C-PC purity by removal of a fraction of small-sized impurities. By using the differences in solubility, a further separation of substances from each other became possible. Fractionation of C-PC by precipitation with ammonium sulfate (AS), at a concentration of 45%, resulted in high- purity C-PC. Ion exchange chromatography was employed for the final purification step to acquire a highly pure form of C-PC protein pigment. Proteins can be efficiently separated by IEX due to plenty of ionizable groups in their molecular structure, creating the diversity in proteins in response to pH (depends on the net charge of the protein) and the particular pattern of charge distribution around the protein surface. Dissimilarity in overall ionic interactions of different proteins can result in differences in their elution through the IEX system. The optimum operating conditions for purification of C-PC in column chromatography have led to remarkable purity index of 5.26. Several previous studies reported the purification of C-PC using practically similar methods, achieving a lower purity level of C-PC compared to the present study^10,11,30–33^. In order to obtain an accurate calculation of the protein purity through the experiments, C-PC solution was diluted whenever required to reduce the absorbance to the lower limit of reliable absorbance spectrophotometer. Purity and recovery amount of phycocyanin through sequential purification steps indicated in Table 4. In a study conducted on the purification of C-PC, this pigment was purified by using ammonium sulfate precipitation and anion exchange chromatography on DEAE-cellulose, achieving ultimate purity index of 4.3^34^. SDS-PAGE analysis revealed that both α and β subunits of the purified C-PC as followed by the present purification protocol have a molecular weight of about 17 and 20 kD, respectively. Correspondingly, in the native gel, the C-PC produced the expected single band with a molecular weight of about 110 kD, indicating that α and β subunits are associated in trimeric form (αβ)3. In a previous study conducted by Gantar *et al*. (2012), the molecular mass of intact C-PC from *Limnothrix* sp. strain 37-2-1 was determined to be ~50 kDa with α and β subunits forming dimmers. The expected molecular mass of the (αβ) monomer was in agreement with the molecular mass and determined to be ~13 kDa for the α-subunit and ~11 kDa for the β-subunit^9^.

**Table 4:** Purity and recovery percent of C-PC.

process	Cell extract	Chitosan	Charcoal	AS	Ion exchange
Purity	1.17	1.49	2.56	3.19	5.26
Recovery %	100	96.4	76.8	68.4	59.1

Over the last few decades, the application of natural products to inhibit carcinogenesis is an important subject of cancer researches due to the toxic side effects of the anticancer drugs. Recent trends of bioactive compounds from cyanobacteria for their potent anti-cancer activity have attracted much attention from the scientific community^9,35^. Several studies have found that C-PC has many useful biological functions including anti-inflammatory and anticancer activities^23,36^. Accordingly, C-PC has potential benefits to be considered as a promising chemotherapeutic agent in the future. Hence, the present study aimed to evaluate the anti-cancer properties of C-PC, as a blue photosynthetic pigment, on human breast cancer cells (MCF-7 cell line). The study of cell viability based on the MTT assay in MCF-7 cells after exposure to the C-PC demonstrated anti-proliferative effects of C-PC on MCF-7 in a dose- and time-dependent manner (Fig. 5a). In a previous report, the inhibitory effects of PC from *cyanobacterium* *Spirulina* on Human ovarian cancer SKOV-3 cells were determined by MTT assay and results showed that the IC50 values of PC were 182.0 μM and 133.6 μM for 24 h and 48 h exposure, respectively^37^. The results obtained in a similar study also demonstrated the anti-tumor effect of PC from *Spirulina platensis* on Human ovarian cancer SKOV-3 cells and the IC50 of the PC was found to be 216.6 and 163.8 μM for 24 and 48 hours, respectively^19^. In a study conducted by Li *et al*. (2015), the effect of the combination of all-trans retinoic acid (ATRA) and C-phycocyanin (C-PC) on the growth and apoptosis of A549 cells was investigated. They have shown both C-PC and ATRA could inhibit the growth of A549 cells *in vivo* and combination of ATRA + C-PC resulted in a higher inhibition rate. C-PC induces a major effect on the proliferation of human embryonic lung cells, but ATRA at a high concentration exerted its effects through inhibition of cells. With an increase in the C-PC and ATRA concentrations, the inhibition rate was boosted and the IC50 of ATRA and C-PC were determined to be 0.204 ± 0.024 mmol/l and 176.62 ± 6.38 μg/l, respectively^38^. The plausible assumptions that may explain the discrepancy between the observed IC50 of C-PC could be due to one of the following reasons: 1) a difference in the sensitivity of distinct cell lines toward the C-PC. 2), a difference in the nature, chemical structure, and the percentage purity of C-PC obtained from various cyanobacterial strains. To assess the conditions that reduce the MCF-7 cell growth, further additional experiments were carried out to reveal the mechanisms underlying the C-PC-induced apoptosis, showing the distribution of MCF-7 cells in different phases of apoptosis. Flow cytometric analysis of cells stained with both PI and Annexin V*-*FITC confirmed that C-PC could induce apoptosis in MCF-7 cells after 24 hours. However, the percentage of early apoptotic, Annexin V-positive and PI-negative, cells decreased over time, leading to late apoptosis or necrosis. There is also a limit that affects the ability to distinguish between late apoptotic and necrotic cells since both of these groups of cells are Annexin V-FITC^+^/PI^+^. Therefore, the rate of apoptosis is just related to the cells which are stained with Annexin V-FITC. In a previous report, the effect of PC, as an anti-neoplastic agent *in vitro* on a series of breast cancer cell lines, were studied and comparable results obtained concerning PC-induced apoptosis in the triple negative breast cancer (MDA-MB-231) cells^35^.

Flow cytometric analysis of cell cycle of MCF-7 cells treated with C-PC, using propidium iodide (PI) DNA staining, showed a significantly induced G2 arrest after 24 hours, while the longer exposures to the lower concentrations of C-PC has resulted in a gradual increase and decrease in the proportion of cells residing in the S and G2 phases, respectively. As it has been noted before, intra-S phase checkpoint delays the cell cycle progression in a discriminative fashion based on the type of DNA damage^39^. It can be speculated that the treatment of MCF-7 cells with the higher concentrations of C-PC resulted in an increased level of cellular reactive oxygen species (ROS), followed by a particular type of DNA damage, which does not invoke the intra-S checkpoint and the passage of the cells through the S phase without a prominent delay. Rhind and Russel (2000) postulated a mechanism for the intra-S checkpoint, describing that the checkpoint slows replication and allows cells to replicate damaged DNA by employing an active repairing system, while replication proceeds at a slower rate^40^. It has been reported that the repairable defects may extend the cells stay in the S phase with a dose-dependent fashion^39^. Accordingly, it is highly likely that prolonged exposure to the lower concentrations of C-PC leads to prolonged DNA damage repair in a large number of cells, resulting in a higher cell population in S phase. Therefore, it can be concluded that C-PC treatment with various exposure time and dosage regimens, may cause several checkpoints to arrest the cell cycle to some extent by a variety of mechanisms. Ying *et al*.^37^ reported that the C-PC-induced G2 cell cycle arrest occurred after 48 hours of incubation and as the concentration of C-PC increases; the induction of G2 cell cycle arrest is further extended^19^. Since mitochondria play a crucial role in activating apoptosis in mammalian cells, we examined the fundamental parameters involved in mitochondrial impairment including ROS production, the collapse of mitochondrial membrane potential (MMP), lipid peroxidation (LPO), ATP level and the GSH and GSSG contents to divulge the possible chain reactions responsible for mitochondrial apoptosis pathway. Results obtained from mitochondrial ROS production and MMP as two important parameters of mitochondrial damage indicated a higher ROS production and decreased MMP level after exposure to C-PC as compared to control cells. The increased mitochondrial ROS formation leads to oxidation of lipids in the membrane, change in the reduced to oxidized glutathione ratios, disruption of mitochondrial electron transport chain (mETC) followed by the collapse of the mitochondrial membrane potential (MMP). Consequently, reducing MMP and rupture of the outer mitochondrial membrane would lead to the opening of mitochondrial permeability transition pores (MPT), the release of cytochrome c and activation of caspase, which ultimately initiates mitochondrial pathway of apoptosis^41–43^. On the other hand, the measurement of ATP indicated a remarkable decrease in the ATP level. It concluded that the opening of the MPT could further result in the decrease in MMP and ATP production^44^. Moreover, a significant reduction in ATP level was observed after 72 hours exposure to C-PC compared to the changes occurred 24 and 48 hours following exposure. Since the level of intracellular ATP is determinant in selection of either apoptosis or necrotic pathways by the stressed cells and based on the results obtained from Annexin V-FITC/PI staining, it seems that cell death occurs by apoptosis at 24 hours in MCF-7 cells^45^. However, as the period of incubation proceeds and cellular ATP levels continue to drop, cell death occurs through a necrotic pathway. Mitochondria are the main source of free radicals and have their own ROS scavenging mechanisms that enriched with many antioxidants such as GSH to minimize oxidative stress damage^46^. The analysis of data for GSH and GSSG contents suggest that C-PC maintains mitochondrial antioxidant capacity within 24 and 48 hours, preventing the oxidation of GSH. However, after 72 hours, the antioxidant properties of C-PC are reduced, resulting in the oxidation of GSH to GSSG. It was previously found that C-PC increases mitochondrial dysfunction by increasing intracellular ROS production, reducing mitochondrial membrane potential, releasing cytochrome C, and eventually activates caspases which lead to the induction of apoptosis^47^. The survey confirmed that C-PC induces apoptosis via the mitochondrial pathway^47^. It seems that C-PC does not have to be target-specific, affecting different parts of cell including nuclear membrane, cytoplasmic membrane as well as mitochondrial membrane. However, the exact mechanism involved in anticancer activity of C-PC has not yet been determined^48,49^. In a recent study conducted by Jiang *et al*. (2018) on human breast cancer cells (MDA-MB-231) treated with C-PC, an important factor associated with cell death was shown to be linked with reduced cyclin D1 expression^49^. Findings from a new study on non-small cell lung cancer (NSCLC) have shown that C-PC could induce apoptosis and cell cycle arrest^6^. It has been revealed that the anti-apoptotic proteins such as *Bcl2* and *Stat3* play a pivotal role in mitochondrial pathway of apoptosis by preventing the release of cytochrome c from mitochondria^50,51^. In addition, *Cyclin D1* is a potential factor in the progression of breast cancer^52^. The impact of C-PC on the expression of *Cyclin D1* in MCF-7 has not yet been investigated. In the present study, the treatment of MCF-7 cells with C-PC downregulated the expression of *Bcl2*, *Stat3* and cyclin in response to C-PC. These findings are in agreement with earlier reports which showed reduced *Cyclin D1* expression in A549 cells after treatment with C-PC^37^. Other studies have also shown that C-PC can induce apoptosis in different cancer cell lines by reducing the expression of anti-apoptotic and increasing the expression of pro-apoptotic proteins^37^.

## Conclusion

Cyanobacteria are supposed to have been one of the most primitive photosynthetic organisms emerged on Earth. C-phycocyanin pigment, as a bioactive compound of phycobiliprotein complexes, possesses a vast range of biotechnological applications particularly in the food, pharmaceuticals, cosmetics, biomedical researches and clinical diagnostics. The present study is the first study of its kind conducted to purify the phycocyanin pigment with a particular emphasis on a novel approach, enhancing its purity index to an appropriate commercial value. No studies have so far been reported concerning mitochondrial functionality during apoptosis and comparison of different types of cell death induced by C-PC in MCF-7 human breast cancer cells over time. Interestingly, our finding clearly indicate that C-PC, in addition to maintaining its antioxidant properties within the first 24 hours of treatment of MCF-7 cells, the highest rate of cell death through apoptosis and a significant cell cycle arrest at G2/M phase occurred at this time. To compare the effect of C-PC on normal and cancer cells, MCF-7 breast cancer cells and human primary fibroblasts treated with C-PC at a concentration of IC50 for every interval (24, 48 and 72 hours), evaluating the possible application of C-PC as an anticancer drug. It was concluded that the treatment of human fibroblast cells with C-PC caused the lowest death rate in a 24-hour period, as over 90% of the primitive fibroblast cells remain alive. However, the percentage of MCF-7 cells undergoing death was about 50%. Consequently, after 24 hours of C-PC treatment compared with 48 and 72 hours, C-PC has the least effect on human fibroblast cells. Several different therapies are now available for treating cancers, but unfortunately, in most cases, the response to treatment is very poor and accompanied by adverse side effects. There is also a marked increase in resistance of cancer cells to existing anticancer drugs. Therefore, research on drug discovery from natural sources for the treatment of different kinds of cancers is of most importance. Since the ultimate goal in treating cancer is to eliminate cancer cells with minimal damage to normal tissues, the induction of apoptosis in cancer cells using microbial natural products can be a good strategy for cancer treatment. In recent decades, apoptosis and the genes that control it have played a significant role in the treatment of cancer. Cyanobacteria are well known as valuable sources of a wide variety of bioactive compounds which have the potential for the induction of apoptosis in various tumor cells. No study has ever been conducted to consider the entire range of mitochondrial changes and to compare the type of cell death induced by C-PC in a broad range of time periods (24, 48 and 72 hours). Therefore, for the first time and based on all the data presented in this paper, it can be assumed that C-PC induces apoptosis in cancer cells due to impaired mitochondrial function. These potential features of C-PC make it a promising drug candidate for further development in cancer treatment.

## Supplementary information

Fig. 3- Sup.
