# Supplementary material for: Development of a novel method for the purification of C-phycocyanin pigment from a local cyanobacterial strain Limnothrix sp. NS01 and evaluation of its anticancer properties
Source: Sci Rep. 2019 Jul 1;9:9474. doi: 10.1038/s41598-019-45905-6 (PMC6603007; doi:10.1038/s41598-019-45905-6)
Supplement: Supplementary file 1 — Fig. 3- Sup. [file 41598_2019_45905_MOESM1_ESM.pdf]

Development of a novel method for the purification of C-phycocyanin pigment from a local cyanobacterial strain Limnothrix sp. NS01 and evaluation of its anticancer properties

Mahdie Safaei[1], Hadi Maleki[2], Hamidreza Soleymanpour[1], Amir Norouzy[1], Hossein Shahbani Zahiri[1], Hojatollah Vali[3], Kambiz Akbari Noghabi[1]*

[1]Department of Energy & Environmental Biotechnology, National Institute of Genetic Engineering and Biotechnology (NIGEB), P.O. Box 14155-6343 Tehran-Iran, [2]Department of Microbiology and Microbial Biotechnology, Faculty of Life Sciences & Biotechnology, Shahid Beheshti University, Tehran-Iran,[3]Facility for Electron Microscopy Research, McGill University, 3640 Street, Montreal, Quebec H3A 0C7 Canada.

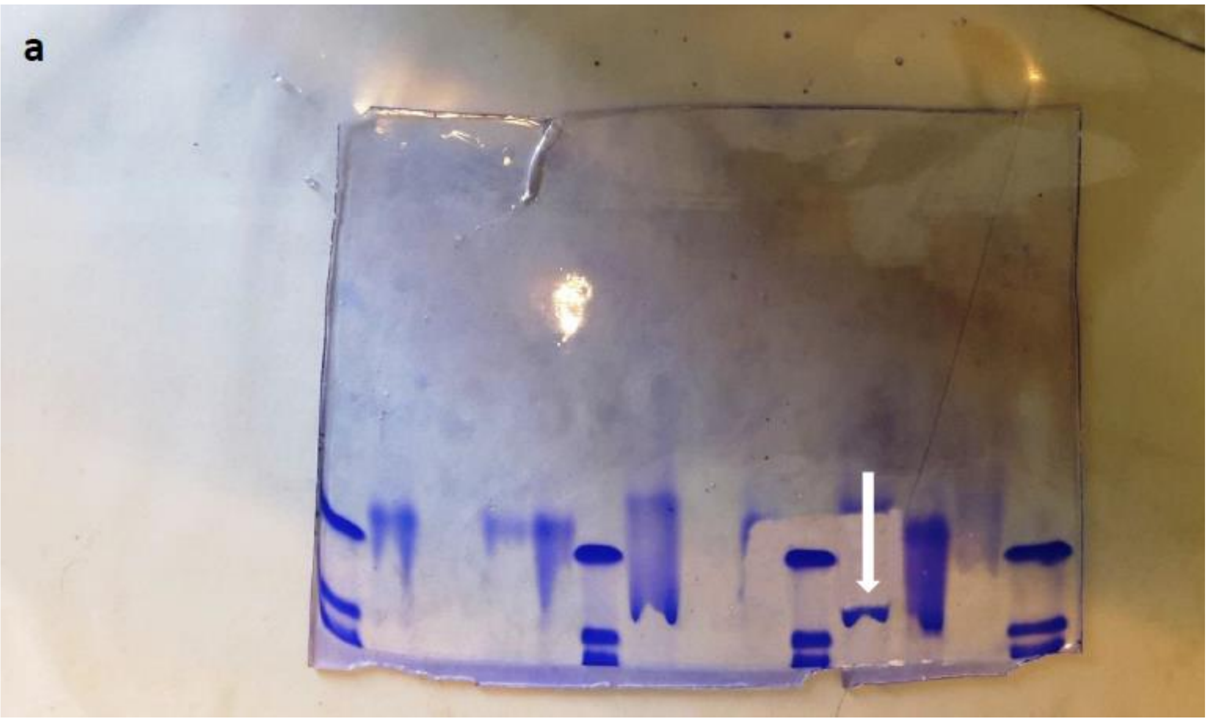

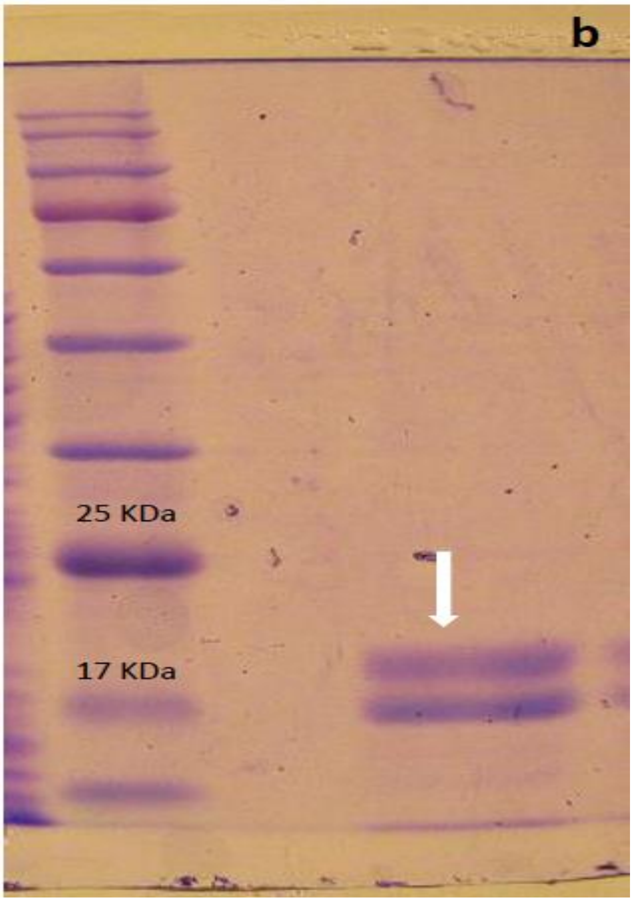
b
25 KDa
17 KDa

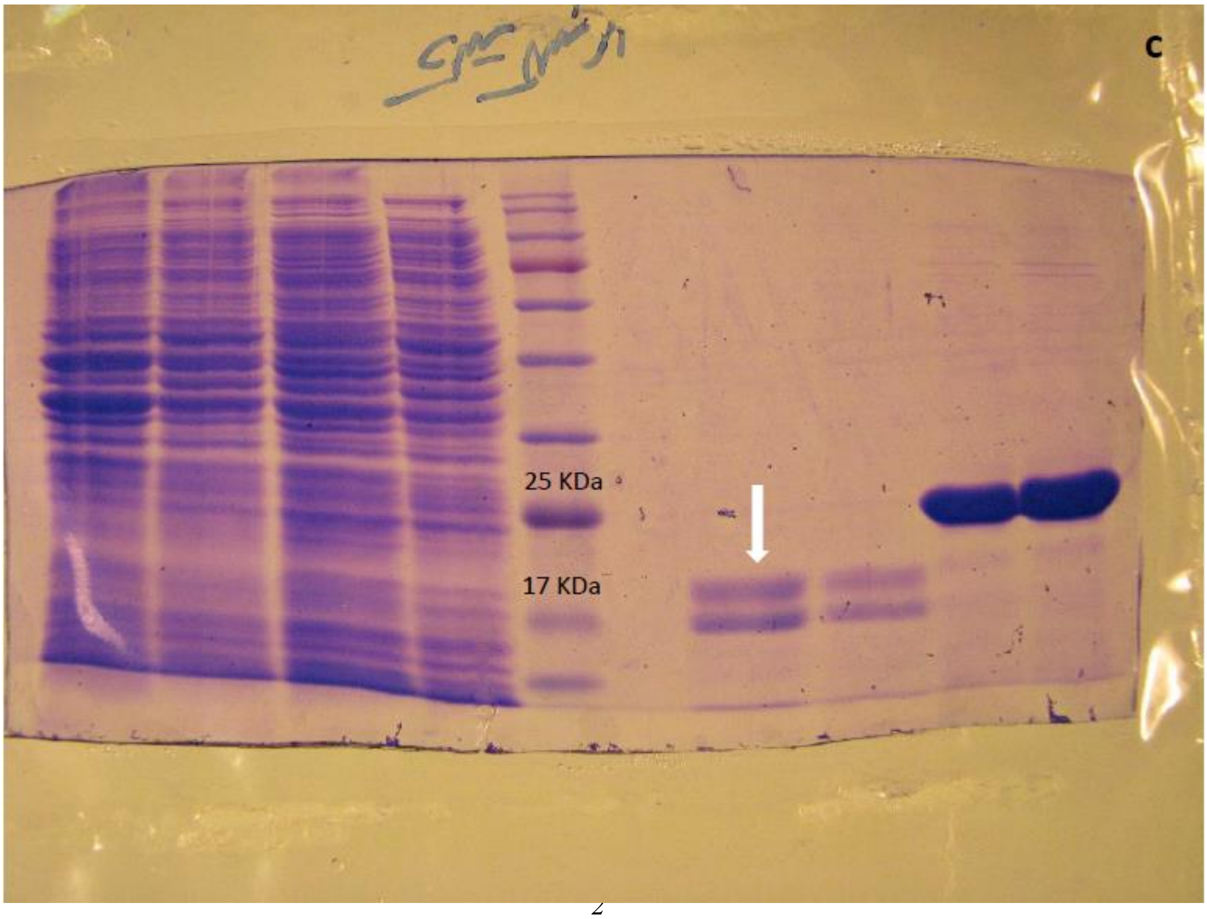
c
25 KDa
17 KDa

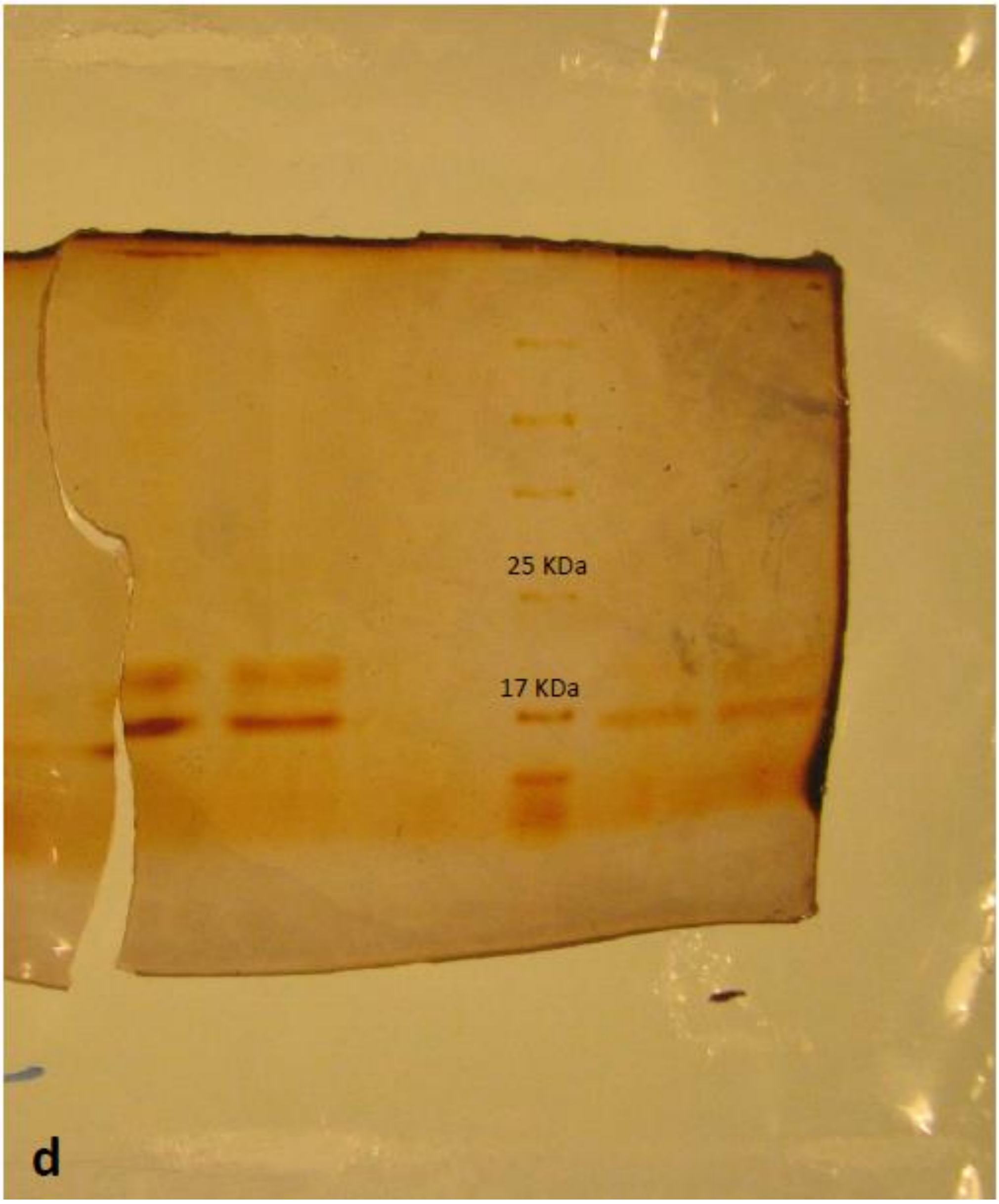

Fig. 3- Sup. Native and SDS-PAGE profiles of the purified form of C-PC from *Limnothrix* sp. NS01. a) Blue-native PAGE of bovine serum albumin (BSA) as a protein marker and single bond of the purified C-PC by Blue-native PAGE. b & c) SDS-PAGE profile of C-PC stained with Coomassie brilliant blue, having a molecular weight of about 17 and 20 kDa, related to the α and ß subunits, d) SDS-PAGE bond of C-PC stained with silver nitrate.
